# TSH-receptor antibodies may prevent bone loss in pre- and postmenopausal women with Graves' disease and Graves' orbitopathy

**DOI:** 10.20945/2359-3997000000027

**Published:** 2018-03-23

**Authors:** Mira Siderova, Kiril Hristozov, Aleksandra Tsukeva

**Affiliations:** 1 University Hospital “St. Marina” Clinic of Endocrinology and Metabolic Diseases Varna Bulgaria Clinic of Endocrinology and Metabolic Diseases, University Hospital “St. Marina”, Varna, Bulgaria; 2 University Hospital “St. Marina” Department of Neurology and Neuroscience Varna Bulgaria Department of Neurology and Neuroscience, University Hospital “St. Marina”, Varna, Bulgaria

**Keywords:** Graves' disease, Graves' orbitopathy, bone mineral density, TSH-receptor antibodies, osteoporosis

## Abstract

**Objective:**

Thyrotoxicosis is established risk factor for osteoporosis due to increased bone turnover. Glucocorticoids often administered for Graves' orbitopathy (GO) have additional negative effect on bone mineral density (BMD). Our aim was to examine the influence of thyroid hormones, TSH, TSH-receptor antibodies (TRAb) and glucocorticoid treatment on bone in women with Graves' thyrotoxicosis and Graves' orbitopathy (GO).

**Subjects and methods:**

Forty seven women with Graves' disease, mean age 55.6 ± 12.8 (23 women with thyrotoxicosis and 24 hyperthyroid with concomitant GO and glucocorticoid therapy) and 40 age-matched healthy female controls were enrolled in the study. We analyzed clinical features, TSH, FT4, FT3, TRAb, TPO antibodies. BMD of lumbar spine and hip was measured by DEXA and 10-year fracture risk was calculated with FRAX tool.

**Results:**

The study showed significantly lower spine and femoral BMD (g/cm^2^) in patients with and without GO compared to controls, as well as significantly higher fracture risk. Comparison between hyperthyroid patients without and with orbitopathy found out significantly lower spine BMD in the first group (p = 0.0049). Negative correlations between FT3 and femoral neck BMD (p = 0.0001), between FT4 and BMD (p = 0.049) and positive between TSH and BMD (p = 0.0001), TRAb and BMD (p = 0.026) were observed. Fracture risk for major fractures and TRAb were negatively associated (p = 0.05). We found negative correlation of BMD to duration of thyrotoxicosis and cumulative steroid dose.

**Conclusions:**

Our results confirm the negative effect of hyperthyroid status on BMD. TRAb, often in high titers in patients with GO, may have protective role for the bone, but further research is needed.

## INTRODUCTION

Thyroid hormones (TH) are one of the major regulators involved in bone metabolism and development among other regulators (1). They play a crucial role in the skeletal growth, peak bone mass acquisition and maintenance of bone mass. Triiodothyronine (T3) plays a primordial role in the skeletal homeostasis and stimulates osteoblast differentiation and activity by complex direct actions on TH-receptors and indirect mechanisms, involving diverse growth factors and cytokines (2). T3 also stimulates osteoclast differentiation and bone resorption, but it still remains unclear whether this effect results from direct action in osteoclasts or indirectly through RANK-RANKL system (3). Thyrotoxicosis is an established risk factor for secondary osteoporosis due to increased bone turnover (4). Glucocorticoids often administered for Graves' orbitopathy (GO) have additional negative impact on bone mineral density (BMD) in these patients by diminishing bone formation.

The circulating antibodies specific to hyperthyroid Graves' disease are directed against the TSH-receptor (receptor for thyroid stimulating hormone) on the cell surface of thyroid follicular cells and behave as thyroid-stimulating antibodies (5). There is now evidence that TSH-receptor is also expressed widely in a variety of extrathyroidal tissues including orbital preadipocyte fibroblasts, playing a pivotal role in the pathogenesis of GO, and bone (6). Recent studies have shown that TSH acts as a direct regulator of bone remodeling through TSH-receptors both on osteoblasts and osteoclasts, highlighting the importance of integrity of the hypothalamo-pituitary-thyroid axis (7). Given that TSH receptor signaling has a functional role in the bone, we could expect that TSH receptor antibodies (TRAb) may also affect bone metabolism. There are conflicting results in the literature regarding the bone effect of TRAb, ranging from bone protection to accelerated bone loss (8–10). Some authors explain this phenomenon in the context of the thyroid status (overt or subclinical hyperthyroidism or restored euthyroidism), others by the type of TRAb (stimulating or blocking) (8–11).

The aim of this study was to examine the influence of TH, thyroid antibodies and glucocorticoid treatment on bone by assessing BMD and fracture risk in women with Graves' thyrotoxicosis alone or with Graves' orbitopathy (GO) and steroid treatment.

## SUBJECTS AND METHODS

Forty seven females with Graves' disease, mean age 55.6 ± 12.8 (23 women with Graves' thyrotoxicosis without orbitopathy and 24 thyrotoxic with concomitant active moderate-to-severe GO and glucocorticoid therapy) were enrolled in the study. Graves' disease was previously diagnosed and mean duration of hyperthyroidism was 43.6 ± 19.2 months in patients without orbitopathy and 32.0 ± 18.5 months in patients with orbitopathy (p = 0.45). At the time of study enrollment 29 patients were in overt hyperthyroidism (16 without orbitopathy and 13 with orbitopathy) and 18 had subclinical hyperthyroidism (7 without and 11 with orbitopathy; p = 0.37). Forty healthy women, mean age 53.2 ± 7.1, without thyroid disease served as age-matched controls. Informed consent was obtained from all patients.

Clinical evaluation including weight, height, body mass index (BMI), cardiovascular parameters, electro-cardiography and thyroid ultrasound were performed. Assessment of GO activity was carried out using the clinical activity score (CAS) consisting of seven items: spontaneous retrobulbar pain, pain on attempted up or down gaze, redness of the eyelids, conjunctival redness, swelling of the eyelids, chemosis, inflammation of the caruncle and/or plica; the final score was calculated as the sum (1 point for each) of all items with CAS ≥ 3/7 points indicating active GO. Severity of GO was assessed according to NOSPECS classification. Data on duration of thyrotoxicosis, duration of GO, cumulative dose and duration of glucocorticoid treatment were collected.

Serum levels of TSH, FT4 (free thyroxine), FT3 (free triiodothyronine), TSH receptor antibodies (TRAb) and TPO antibodies were analyzed through chemiluminescent immunoassays (Siemens Healthcare Diagnostics) on ADVIA Centaur XP and Immulite 2000 systems with following reference ranges: TSH 0,4-4,0 mU/l; FT4 10,3-24,0 pmol/l; FT3 2,8-6,5 pmol/l; TRAb with normal range < 1IU/l and TPO-Ab 10-100 U/mL.

BMD of total hip, femoral neck and lumbar spine (L1-L4) was measured by GE-Lunar Prodigy DEXA and the 10-year fracture risk was calculated with FRAX tool (https://www.shef.ac.uk/FRAX/tool.jsp). This was first BMD measurement for all participants, so they could not provide any information of previous BMD or bone metabolic markers. Neither patients, nor controls were taking calcium or vitamin D3 supplementation.

Statistical analysis of the data was performed by SPSS v.19.0 (SPSS, Chicago, USA). The data are expressed as mean ± standard deviation (SD). Student's T-test was used to compare continuous variables. Non-metric variables were presented by relative frequency distribution (in percentages). Fisher's exact test was used to detect differences between the groups. The Spearman correlation method was performed between different measured parameters. A p-value (two-tailed) of less than 0.05 was considered statistically significant with statistical power 80% for the sample size.

## RESULTS

Patients and controls did not differ for age, prevalence of smokers and premenopausal women. Four women of the patients' group had fractures (2 with and 2 without steroid treatment). Both patients on steroid treatment had a vertebral fracture, whereas one patient without steroid treatment had a vertebral fracture and the other had a distal radius fracture. Although there was no significant difference in BMI (25.3 ± 3.23 vs 26.1 ± 3.53, p = 0.29), the body weight was lower in the patients' group (64.8 ± 7.9 vs 69.1 ± 9.2, p = 0.025), but weight loss is a well known clinical characteristic of Graves' disease. The study showed significantly lower BMD (g/cm^2^) at spine, total hip and femoral neck in patients compared to healthy controls (p < 0.0001; p < 0.0001; p = 0.0002). The results are summarized in Table 1.

**Table 1:** Comparison between clinical characteristics, laboratory exams, DEXA and FRAX assessment in patients and controls

	Patients (Thyrotoxicosis with/without GO)	Healthy controls	p-value	
Number	47	40		
Mean age (years)	55.6 ± 12.8	53.2 ± 7.1	p = 0.25	NS
Weight (kg)	64.8 ± 7.9	69.1 ± 9.2	p = 0.025*	S
BMI (kg/m^2^)	25.3 ± 3.23	26.1 ± 3.53	p = 0.29	NS
TSH (mU/l)	0.0185 ± 0.13	1.840 ± 0.96	p < 0.001**	S
TRAb (IU/l)	16.2 ± 13.32	Negative (< 1.0)	p < 0.0001***	S
Women in menopause	36/47	31/41	p = 0.327	NS
Mean age of menopause	47.7	48.6	p = 0.391	NS
BMD spine (g/cm^2^)	0.994 ± 0.15	1.197 ± 0.08	p < 0.0001***	S
BMD total hip (g/cm^2^)	0.885 ± 0.12	0.999 ± 0.07	p < 0.0001***	S
BMD fem. neck (g/cm^2^)	0.845 ± 0.11	0.920 ± 0.06	p = 0.0002***	S
FRAX (major osteoporotic fractures %)	6.85	3.49	p < 0.0001***	S
FRAX (hip fractures %)	1.95	0.36	p < 0.0001***	S

NS: not significant, p > 0.05;

* /S: significant; p = 0.01-0.05;

** very significant, p = 0.001-0.01

*** extremely significant, p < 0.001.

Both subgroups of patients with and without GO had lower BMD than controls (p = 0.0002; p < 0.0001), as well as significantly higher 10-year fracture risk for major osteoporotic and hip fractures (p < 0.0001; p < 0.0001).

Comparison between patients with Graves' thyrotoxicosis without orbitopathy and those with additional steroid-treated Graves' orbitopathy found out significantly lower spine BMD in the first subgroup (p = 0.0049). However, the duration of thyrotoxicosis in this first subgroup was longer (43.6 vs 32 months, p = 0.45). The GO subgroup was younger than the subgroup without orbitopathy, but this diffenrence did not reach statistical significance (52.6 ± 12.3 years vs. 58.5 ± 11.9 years, p = 0.14). Although the study population was heterogeneous concerning menopausal status, there was no significant difference in number of postmenopausal women between the subgroup without GO and that with GO (20/23 vs 16/24, p = 0.17). Table 2 presents the analysis of both subgroups of hyperthyroid patients.

**Table 2:** Comparison between clinical characteristics, laboratory exams, DEXA and FRAX assessment in Graves' patients with and without Graves' orbitopathy

	Graves' disease without orbitopathy	Graves' disease with orbitopathy (GO)	p-value	
Number	23	24		
Mean age (years)	58.5 ± 11.9	52.6 ± 12.3	p = 0.14	NS
Women in menopause	20/23	16/24	p = 0.17	NS
Duration of hyperthyroidism (months)	43.6 ± 19.2	32.0 ± 18.5	p = 0.45	NS
TSH (mUI/l)	0.017 ± 0.12	0.019 ± 0.14	p = 0.62	NS
TRAb (IU/l)	15.58 ± 13.0	16.61 ± 13.5	p = 0.88	NS
BMD spine (g/cm^2^)	0.936 ± 0.14	1.059 ± 0.15	p = 0.0049**	S
BMD total hip (g/cm^2^)	0.863 ± 0.11	0.9123 ± 0.12	p = 0.205	NS
BMD fem. neck (g/cm^2^)	0.836 ± 0.11	0.858 ± 0.11	p = 0.517	NS
FRAX (major osteoporotic fractures %)	5.97	7.91	p = 0.200	NS
FRAX (hip fractures %)	1.64	2.32	p = 0.313	NS

NS: not significant, p > 0.05.

** very significant, p = 0.001-0.01.

In patients' group as a whole we observed negative correlation between FT3 and BMD (p = 0.019 for spine; p = 0.0001 for total hip and p = 0.0001 for femoral neck), as well as between FT4 and BMD of femoral neck (p = 0.049). Data are presented in Table 3. There was a positive association between TSH and BMD with statistical significance for total hip and femoral neck (p = 0.0001; p = 0.0001). We found also significant positive correlation between TRAb and BMD (p = 0.006 for spine; p = 0.013 for total hip and p = 0.026 for femoral neck) and a negative correlation between TRAb and fracture risk for major fractures (p = 0.050). The analysis showed negative association of BMD to duration of steroid treatment for GO and cumulative steroid dose with significance for total hip (p = 0.016; p = 0.030) and femoral neck (p = 0.011; p = 0.020). There was also negative correlation between BMD and duration of thyrotoxicosis (p = 0.048 for spine; p = 0.045 for total hip and p = 0.040 for femoral neck).

**Table 3:** Correlations between clinico-laboratory parameters and BMD/fracture risk

	BMD Spine	BMD total hip	BMD femoral neck	FRAX major osteoporotic fractures	FRAX hip fractures
FT3	r −0.361	r −0.635	r −0.665	r 0.262	r 0.267
	p 0.019	p 0.0001	p 0.0001	p 0.117	p 0.110
FT4	r −0.021	r −0.100	r −0.250	r 0.037	r 0.129
	p 0.807	p 0.439	p 0.049	p 0.817	p 0.416
TSH	r 0.120	r 0.418	r 0.539	r −0.045	r −0.263
	p 0.282	p 0.0001	p 0.0001	p 0.688	p 0.017
TRAb	r 0.412	r 0.380	r 0.348	r −0.292	r −0.231
	p 0.006	p 0.013	p 0.026	p 0.050	p 0.07
TPOAb	r −0.357	r −0.421	r −0.434	r 0.229	r −0.115
	p 0.024	p 0.007	p 0.005	p 0.155	p 0.481
Duration of steroid treatment for GO	r −0.434	r −0.557	r −0.585	r 0.371	r −0.05
	p 0.072	p 0.016	p 0.011	p 0.129	p 0.844
Cumulative dose of steroids	r −0.402	r −0.515	r −0.541	r 0.397	r 0.051
	p 0.100	p 0.030	p 0.020	p 0.103	p 0.841

## DISCUSSION

Thyrotoxicosis is a well-established cause for high bone turnover osteoporosis and fragility fractures (12). Our study demonstrates lower bone mineral density at axial skeleton (spine and hip) in hyperthyroid perimenopausal women with Graves' disease compared to healthy controls in accordance with results of many other authors (12,13). Some studies have proved that in premenopausal women this decrease in the bone density is observed mainly in cortical bone (femoral neck) similarly to other examples of secondary osteoporosis (14). Histomorphometric analysis has shown that thyrotoxicosis results in an increased frequency of bone remodelling cycle initiation and a shortened cycle duration (15). The bone formation phase is reduced to a greater extent than the resorption phase, leading to a 10% loss of bone per cycle (15,16).

Our results showed unexpectedly lower spine BMD in the subgroup of Graves' patients without orbitopathy than that of the subgroup with GO, despite the steroid treatment in the latter. This phenomenon could be partially explained with the longer duration of thyrotoxicosis in the subgroup of patients without GO. The difference in age and menopausal status was not significant between the two subgroups to affect the findings at lumbar spine BMD.

Another factor influencing the difference in BMD between hyperthyroid patients with and without GO is the short duration and low cumulative dose of steroid treatment in GO subgroup. Since we aimed to include patients in the study and to perform DEXA scan as soon as possible, the cumulative dose of already started steroid treatment was relatively low (2050 ± 1200 mg) as well as duration of steroid treatment for GO was relatively short (3,1 ± 1.9 months). This confounding factor could also be the reason for the insignificant correlation between cumulative steroid dose/duration of steroid treatment and spine BMD in our study. The main effect of glucocorticoids on bone is inhibition of osteoblast function, leading to a decrease in bone formation (17). Several studies and reports show a decrease in bone mineral density and an increased risk of fractures during glucocorticoid use for GO (18). Bone loss starts promptly after initiation of glucocorticoids and is mainly taking place in the first six months of treatment (17).

Last but not least both TSH and TRAb were higher in the subgroup of GO patients although this difference did not reached statistical significance. Physiologically TSH stimulates T4 secretion by thyroid follicular cells (13,19), however, the effects of the hypothalamic–pituitary–thyroid (HPT) axis on its target organs are related to levels of TH and to the activities of the deiodinase enzymes that regulate the local concentration of triiodothyronine (T3). Thus, T4 is a pro-hormone of the biologically active T3 (19,20). A recent study on human osteoblast cell line showed increased expression of deiodinase 2 (which converts T4 to T3) by administration of TSH, suggesting that TSH may indirectly promote bone turnover by increasing local T3 availability (21). Furthermore, the HPT axis set-point in an individual is at least in part genetically determined and may influence fracture risk (22,23).

Our results confirmed a negative correlation between FT3 and BMD at spine, total hip and femoral neck), as well as between FT4 and BMD of femoral neck. In the studies of van Rijn and cols. similar association was found in euthyroid perimenopausal women – higher fT4 levels within the normal reference range were independently related to decreased BMD at lumbar spine (13).

It is well established that thyroid hormones (TH), acting via thyroid receptors alpha (TRα), promote catabolic actions on the adult skeleton (19,24). Some authors have proposed that TSH plays important role in bone tissue, which is independent of the actions of TH (7,25,26). This is supported by studies on mice with deletion of the TSHR gene, which show high TSH and low serum levels of TH. TH therapy in these mice affect body weight but not bone mass (7,27,28). Another study has suggested that TSH binding to its receptor in bone cells has beneficial effects on the skeleton through inhibition of osteoclastogenesis (29).

In the study of Tuchendler and Bolanowski BMD changes among premenopausal women with newly diagnosed hyperthyroidism before and after reaching normal thyroid function were assessed (14). There was a clear decrease in the BMD at femoral neck of hyperthyroid patients compared to controls, as well as higher serum concentrations of osteocalcin and collagen type 1 crosslinked C-telopeptide (14). Despite the increase in BMD after one year of methimazol treatment and restoration of euthyroidism, significantly lower femoral neck BMD was observed in patients compared to controls, indicating that correcting the hormonal dysfunction does not fully normalize bone density (14). Probably the effect of TSH which is the last to normalize and the TSH-Ab is responsible for these observations. Another study of Haras and cols. proved that TSH raise during treatment of hyperthyroid patients is the best predictor for bone density increase (30).

Our data revealed a positive significant correlation between TSH and BMD, as well as between TRAb and BMD, and a negative association between TRAb and fracture risk. Similarly, Ma and cols. suggested that in Graves' disease the antibody TRAb decreases bone loss against the catabolic effects of high circulatory levels of TH (8). According to them, TSHR-Abs offer skeletal protection in hyperthyroid Graves' disease, even in the face of high thyroid hormone and low TSH levels (8). By contrast, in Graves' patients with subclinical hyperthyroidism with normal FT4 and FT3, TRAb were positively associated with bone metabolic markers, including B-ALP, U-PYD, and U-DPD (9). In the study of Ecolano and cols. patients with previous Graves' disease hyperthyroidism and restored euthyroidism for at least 6 months were investigated (10). In postmenopausal women BMD of lumbar spine correlated negatively with TRAb and positively with the time of euthyroidism, but neither with serum T4 nor TSH (10). In a multiple regression analysis TRAb was the only significant independent variable in relation to lumbar spine BMD, which suggest that this antibody may affect bone metabolism (10). However, discrepancies exist in the results regarding the role of TRAb in bone metabolism. They could be summarized as bone protective role in the settings of overt hyperthyroidism (8) and accelerated bone loss in subclinical hyperthyroidism and euthyroidism (9,10) underlining the complex interactions of TH, TSH and TSH-R antibodies locally in the bone. Further studies are needed to elucidate their role, as well as the role of inhibiting TSH-R antibodies for bone metabolism.

The strength of the current study is that all participants were peri-menopausal women within a narrow age range and they were not receiving hormone replacement therapy, neither calcium, vitamin D or anti-osteoporosis medication. However, several limitations of our study should be considered. First, DEXA scan was performed shortly after the start of steroid treatment in the group of patients with GO, which can not segregate the effect of steroids from the influence of TH, TSH and TRAb on bone metabolism. Second, bone metabolic markers were not measured in this study and they could add additional information to BMD conserning the effect of hypothalamic–pituitary–thyroid axis and thyroid antibodies on skeleton. Third disadvantage is the lack of follow up and assessment of BMD after restoration of euthyroidism, especially in the cases that will appear with low or negative TRAb in the follow up.

In conclusion, our results confirm the negative effect of hyperthyroid status on BMD. Whereas TH are negatively correlated with BMD, TSH has a positive influence. TSH-receptor antibodies, often in higher titers in patients with Graves' orbitopathy, might have protective role for the bone, but further research is needed.
